# Decentralized, Community-Based Hepatitis C Point-of-Care Testing and Direct-Acting Antiviral Treatment for People Who Inject Drugs and the General Population in Myanmar: Protocol for a Feasibility Study

**DOI:** 10.2196/16863

**Published:** 2020-07-14

**Authors:** Bridget Louise Draper, Alisa Pedrana, Jessica Howell, Win Lei Yee, Hla Htay, Khin Sanda Aung, Sonjelle Shilton, Khin Pyone Kyi, Win Naing, Margaret Hellard

**Affiliations:** 1 Disease Elimination Burnet Institute Melbourne Australia; 2 School of Public Health and Preventive Medicine Monash University Melbourne Australia; 3 Consultant Gastroenterologist St Vincent's Hospital Melbourne Melbourne Australia; 4 Department of Medicine University of Melbourne Melbourne Australia; 5 Burnet Institute Myanmar Yangon Myanmar; 6 Myanmar National Hepatitis Control Program Naypyidaw Myanmar; 7 Foundation for Innovative New Diagnostics Geneva Switzerland; 8 Myanmar Liver Foundation Yangon Myanmar; 9 Yangon Specialty Hospital Yangon Myanmar; 10 University Of Medicine (1) Yangon Myanmar; 11 Department of Infectious Diseases The Alfred Hospital Melbourne Australia; 12 Doherty Institute and School of Population and Global Health University of Melbourne Melbourne Australia

**Keywords:** hepatitis C, primary health care, community health services, delivery of health care, point-of-care testing, retention in care, Asia, Southeastern

## Abstract

**Background:**

The advent of direct-acting antivirals (DAAs) and point-of-care (POC) testing platforms for hepatitis C allow for the decentralization of care to primary care settings. In many countries, access to DAAs is generally limited to tertiary hospitals, with limited published research documenting decentralized models of care in low-and middle-income settings.

**Objective:**

This study aims to assess the feasibility, acceptability, effectiveness, and cost-effectiveness of decentralized community-based POC testing and DAA therapy for hepatitis C among people who inject drugs and the general population in Yangon, Myanmar.

**Methods:**

Rapid diagnostic tests for anti-hepatitis C antibodies were carried out on-site and, if reactive, were followed by POC GeneXpert hepatitis C RNA polymerase chain reaction tests. External laboratory blood tests to exclude other major health issues were undertaken. Results were given to participants at their next appointment, with the participants commencing DAA therapy that day if a specialist review was not required. Standard clinical data were collected, and the participants completed behavioral questionnaires. The primary outcome measures are the proportion of participants receiving GeneXpert hepatitis C RNA test, the proportion of participants commencing DAA therapy, the proportion of participants completing DAA therapy, and the proportion of participants achieving sustained virological response 12 weeks after completing DAA therapy.

**Results:**

Recruitment was completed on September 30, 2019. Monitoring visits and treatment outcome visits are scheduled to continue until June 2020.

**Conclusions:**

This feasibility study in Myanmar contributes to the evidence gap for community-based hepatitis C care in low- and middle-income settings. Evidence from this study will inform the scale-up of hepatitis C treatment programs in Myanmar and globally.

## Introduction

Globally, an estimated 71 million people are living with hepatitis C virus infection [1]. Left untreated, hepatitis C can lead to complications, including cirrhosis, liver failure, and hepatocellular carcinoma [2]. The advent of direct-acting antivirals (DAAs) revolutionized hepatitis C treatment [3-6]. DAAs have few side effects, involve an all-oral regimen, and most people require treatment for only 8-12 weeks [7]. Given that hepatitis C treatment is now simple and safe, there is a push to simplify clinical care pathways to enable rapid scale-up and access to treatments. Although the advent of DAAs made decentralized models of care led by general practitioners (GPs) in primary care settings possible [8,9], the dominant model remains as specialist physicians prescribing at tertiary hospitals. Alongside the introduction of these new treatments, there are various World Health Organization (WHO) prequalified point-of-care (POC) testing and diagnostic technologies available for hepatitis C [10,11]. The availability of POC testing technologies means same-day on-site diagnosis is now possible, reducing the number of times a participant needs to attend the clinic to receive a diagnosis and initiate treatment [7].

Decentralized testing and treatment approaches are supported by the WHO Guidelines for the Care and Treatment of Persons Diagnosed with Chronic Hepatitis C Virus Infection (July 2018) [7], specifically at primary health facilities or harm reduction sites, to promote access to care. These models of care can be implemented using a differentiated care approach that allows for specialist referral as required [7]. Implementation of this model requires simplified diagnostic pathways, training of GPs, specified referral pathways for complicated cases, and access to DAAs in community settings. In this paper, we have defined GPs as medical doctors with no further specialized training after completing their university qualifications. They are sometimes referred to as generalist doctors or primary care physicians in other settings, although it should be noted that in many settings, primary care physicians/GPs often have specialized training. Globally, research trials and programs led by non-government organizations have included decentralized community-based, GP-led models of care [12,13] and, more recently, trials of same-day treatment initiation models [12,14]. However, there is little well-documented evidence of these models of care and their feasibility and effectiveness, particularly in resource-limited settings [12].

In the Republic of the Union of Myanmar (hereafter, Myanmar), an estimated 1.4 million people have hepatitis C infection, and the estimated overall population prevalence is 2.7% [15]. Similar to many resource-limited settings, it is likely that most people acquired hepatitis C in official and nonofficial health care settings, including via unsafe surgery, contaminated blood transfusions, repeated use of syringes for vaccinations/injections, or unsafe dental treatment [7]. At the same time, there is a substantial level of infection among people who inject drugs (PWID), with an estimated 56% prevalence across Myanmar, reaching 84% in some regions [16]. The likely mode of acquisition in this population is through shared injecting equipment.

Currently, in Myanmar, DAAs are mostly available through the National Hepatitis Control Program in tertiary hospitals, the private sector, some international and local nongovernmental organizations, and research projects (generally focusing on people with HIV and hepatitis C co-infection). There is currently limited capacity in centralized laboratories in Myanmar to provide hepatitis C RNA polymerase chain reaction (PCR) testing, other than those utilizing the Cepheid GeneXpert platform.

Given the large numbers of people infected, if treatment is to be accessible to everyone with hepatitis C infection, it is important that treatment is available in both community settings and tertiary hospital settings. Few countries allow GPs to prescribe DAA therapy [17], and Myanmar is one of them [18]. The National Guidelines for the Simplified Treatment of Hepatitis C Virus Infection allow GPs to prescribe DAA therapy and for the use of the Cepheid GeneXpert hepatitis C RNA PCR test to assess if someone has current hepatitis C infection [18]. However, to date, there is insufficient data to assess the success of this decentralized approach, including the use of the community-based diagnostic and treatment pathway in Myanmar and other low-and middle-income settings globally [12,14,19].

The Hepatitis C: Community-based Testing and Treatment Study (CT2 study), undertaken by the Burnet Institute, is part of the Foundation for Innovative New Diagnostics (FIND)–led Hepatitis C Elimination through Access to Diagnostics (HEAD-Start) program funded by Unitaid. It was designed to assess the feasibility of a decentralized hepatitis C model of care in a resource-constrained setting, utilizing POC testing technologies with a GP initiating DAA therapy in the majority of participants, with a pathway of care to tertiary hospitals for people with decompensated cirrhosis. This paper describes the protocol of the CT2 study.

## Methods

### Study Design

The CT2 study is a feasibility trial of decentralized community-based hepatitis C testing utilizing POC testing technologies and GP-led DAA treatment initiation and measures on the outcomes of completion of the hepatitis C diagnostic pathway, treatment initiation, and treatment outcomes. The study aimed to treat 450 participants for hepatitis C infection with the pangenotypic regimen sofosbuvir 400 mg and daclatasvir 60 mg.

The study protocol (version 7, June 27, 2019) follows the Standard Protocol Items: Recommendations for Interventional Trials statement. The trial was registered at ClinicalTrials.gov NCT03939013 on May 6, 2019.

### Trial Status

Recruitment commenced on January 30, 2019 and was completed on September 30, 2019.

### Objectives

This study will determine if implementing this model of care is feasible in Myanmar. It assesses the key requirements for implementation in a resource-constrained setting, the acceptability of the model of care to participants and providers, and the cost of implementing the model in this setting.

#### Primary Outcome Measures

The primary outcome measures include:

1. The proportion of anti-hepatitis C antibody (Ab)-positive participants who receive a GeneXpert hepatitis C RNA PCR test
2. The proportion of hepatitis C RNA PCR-positive participants who are initiated on DAAs
3. The proportion of participants initiated on treatment who complete therapy (defined as picking up the last 28-day bottle of medication and not reporting >7 days missed doses)
4. The proportion of participants who completed treatment who achieve a sustained virologic response (SVR12).

#### Secondary Outcome Measures

The secondary outcome measures include:

1. The proportion of participants requiring specialist review
2. Feasibility of the testing and treatment pathways from the provider perspective
3. Acceptability of the testing and treatment pathways participant
4. Costing of POC testing and treatment pathways at community sites
5. Time from the collection of hepatitis C RNA test samples to treatment initiation.

#### Feasibility Assessment

To assess the feasibility of this model of care, we will focus on 3 of the domains identified by the study by Bowen et al [20]—*How We Design Feasibility Studies*: demand, implementation, and practicality. Demand will be assessed using testing and treatment uptake (primary outcome measures number 1 and number 2). Implementation and practicality will be assessed using the Gericke et al [21] *Intervention Complexity* framework: (1) characteristics of basic intervention, (2) characteristics of delivery, (3) requirements on government capacity, and (4) usage characteristics. For these dimensions, we will focus on the requirements for the GeneXpert device and GP-initiated DAAs.

### Study Setting and Recruitment

#### Study Setting

This study is being conducted in Yangon, Myanmar. Yangon is the largest city in Myanmar, with a population of over 7 million people [22]. Yangon has five hospital sites (three for HIV/hepatitis C co-infected patients, two for hepatitis C mono-infected patients) that can provide hepatitis C treatment at no cost as part of the government-led National Hepatitis Control QuickStart program; a subset of hospitals also provide a subsidized treatment program to patients. However, only a small proportion of patients requiring treatment can currently access treatment through the government-led program. As a consequence, there is a high unmet need for no-cost/low-cost hepatitis C testing and treatment programs among the general population in Yangon as well as for PWID, who are often unable to access hospital-based programs.

#### Study Sites

The study is being conducted at 2 community clinics in Yangon: the Burnet Institute Thingangyun Key Population Service Clinic (hereafter referred to as the Burnet clinic) and the Myanmar Liver Foundation (MLF) Than Sitt Charity Clinic (hereafter referred to as the MLF clinic). Clinics are staffed by a trained GP (study medical officer), a nurse, and a laboratory technician for the duration of the study, plus a peer worker at the Burnet clinic and reception staff at MLF clinic. These study sites are separate from the hospital program sites described earlier.

The Burnet clinic provides health care services primarily to PWID, including needle and syringe distribution. The Burnet clinic is located in the Thingangyun township near the major methadone treatment center in central Yangon.

The MLF clinic serves the general population of patients with liver disease, with a focus on hepatitis B and hepatitis C care. MLF is well known across Myanmar for providing hepatitis B vaccination and treatment and hepatitis C testing and treatment; it runs several clinics across the country. MLF clinics provide outreach hepatitis C screening to various townships on an ad hoc basis, with those who test positive referred to MLF clinics for further testing and, if available, treatment through research studies, philanthropic projects, and other cost-sharing arrangements. The MLF clinic in Yangon also advertises its services via Facebook and accepts referrals from physicians for patients with elevated liver enzymes and other indicators of hepatitis B or hepatitis C.

#### Recruitment

Recruitment into the study was based on prescreening eligibility assessment (criteria described further in *Study Assessments, Pretreatment Assessments* section). At the Burnet clinic, the study was advertised to potential participants using flyers displayed at the methadone treatment centers in Yangon and through Burnet peer outreach workers distributing flyers and speaking with PWID about the study. Burnet peer outreach workers also assessed potential participants for eligibility for enrollment in the study and made initial screening appointments with the nurse and medical officer at the clinic.

MLF clinic medical officers registered potential participants (including those previously screened anti-hepatitis C Ab positive) before commencement of the study and then invited them to attend the clinic in order of registration date. Patients attending the clinic on specified recruitment days (3 to 5 days per week) were also offered participation in the study.

At the initial study visit, the study nurse explained the purpose and procedure of the study to interested participants. Pre-enrollment eligibility was then assessed by the study nurse, and the consent procedure was conducted by the study nurse and medical officer.

Study recruitment commenced at the MLF clinic on January 30, 2019, and at the Burnet clinic on February 4, 2019; the last participant was recruited on September 30, 2019. Each clinic aimed to start 225 participants on DAA therapy; a total of 585 3-month treatment courses were available for the study, including some 6-month courses allocated to treat cirrhotic participants.

#### Eligibility

Before enrollment, participants had to be:

1. Aged 18 years or older
2. Willing and able to provide written informed consent
3. Not previously tested for hepatitis C virus (HCV) RNA
4. Hepatitis C treatment naïve (no prior pegylated interferon-based therapy or DAA therapy)
5. Without HIV or hepatitis B infection and active tuberculosis/being treated for tuberculosis, with known estimated glomerular filtration rate (eGFR) <30 mL/min/1.73m^2^ and not pregnant (all self-reported)
6. Taking no medications with serious drug-drug interactions with sofosbuvir/daclatasvir that the participant would be unable/unwilling to cease.

Eligible participants were consented by delegated study staff (nurse and medical officer), provided written informed consent, and then study procedures commenced.

### Study Interventions

#### Point-of-Care Anti-Hepatitis C Antibody Test

Trained laboratory technicians performed phlebotomy and conducted a POC immunochromatographic rapid test for anti-hepatitis C antibodies, using whole blood venous samples. This study used the WHO-prequalified SD BIOLINE hepatitis C test (Standard Diagnostics Inc, South Korea). It has 99.3% (95% CI 97.9%-99.8%) sensitivity and 100% (95% CI 99.7%-100%) specificity [23], with the result read within 5 to 20 min of the test start time.

The results were then communicated to the medical officer using internal laboratory test forms filled out by the laboratory technician; the medical officer returned the test results to the participants and provided relevant posttest counseling. If reactive, the participants were offered a near-POC hepatitis C RNA test using the remaining blood sample to determine if they had active hepatitis C infection.

#### Point-of-Care Hepatitis C RNA Test

Participants who were anti-hepatitis C Ab positive underwent a near-POC hepatitis C RNA test at the study site (Cepheid GeneXpert Hepatitis C RNA test). The WHO-prequalified GeneXpert hepatitis C RNA test is a reverse transcriptase PCR test performed on blood plasma samples using fluorescence to detect RNA; it is designed to quantify the viral load of hepatitis C RNA [10]. Its sensitivity was 100% (95% CI 94.6%-99.9%) and specificity was 100% (95% CI 75.9%-99.4%); the device error rate was 1.67% [10].

The blood sample was first spun in a centrifuge at 1500 rpm for 10 to 15 min (as per the manufacturer’s instructions) to separate plasma from the whole blood sample, within 2 hours of sample collection. The plasma was then pipetted into the GeneXpert cartridge, and the cartridge was placed in an available module in the GeneXpert machine, the module cover was closed, and the assay commenced; the run time was 105 minutes. Tests were not batched, so they can be performed individually without delay.

GeneXpert results can be hepatitis C RNA detected (within the quantitative range, above the quantitative range [>1.00E08 IU/mL] or below the quantitative range [<10 IU/mL]) or hepatitis C RNA not detected. If the result is RNA detected, but below the quantitative range, the participant will be retested in 12 weeks. If the result is RNA detected and within or above the quantitative range, then the participant is considered to currently have hepatitis C infection.

Participants with an RNA-detected result then have an HIV antibody (Ab) rapid test and hepatitis B surface antigen (HBsAg) rapid test; if both these tests are negative, blood samples are sent to an external laboratory for standard pretreatment laboratory investigations (refer to the *Pretreatment Assessments* section). If the HBsAg test is positive, hepatitis B surface antibody (HBsAb) and hepatitis B core antibody (HBcAb) tests were performed at the laboratory. If the HIV Ab rapid test was s positive, the participant was referred for confirmatory testing and withdrawn from the study if HIV positive.

Generally, all blood required for diagnostic testing and pretreatment assessments was collected in one blood draw.

#### Direct-Acting Antiviral Therapy

Participants who tested RNA detected (or positive) in the study could then receive a combination DAA therapy of sofosbuvir (400 mg) and daclatasvir (60 mg). Those with an aspartate aminotransferase to platelet ratio index (APRI) <2.0 (noncirrhotic) were offered a 12-week course. Those with an APRI ≥ 2.0 (cirrhotic) were offered a 24-week course, consistent with the national Simplified Treatment Guidelines for Hepatitis C Infection. A previous APRI validation study found that an APRI <2.0 reliably excluded cirrhosis in 85% of patients, with a negative predictive value of 91% [24]. (In line with the updated National Guidelines [second edition, July 18, 2019], the APRI cutoff was changed to 1.5 from August 6, 2019.)

All participants were reviewed and initiated on therapy by the study medical officer (a trained GP) at the study site. A specialist review was only required for those with signs of liver decompensation; the study medical officer referred to a consulting hepatologist as necessary and to other specialists at the local tertiary hospital at his or her discretion for other major comorbidities. A total of 4 weeks of medication is dispensed at the study site by the medical officer or nurse (site dependent) at baseline, week 4, and week 8 of treatment and at weeks 12, 16, and 20 for those on a 24-week course. Participants self-administer the combination therapy daily; adherence is monitored using self-report and pill counting if the participants return medication bottles.

The exclusion criteria for commencing DAA therapy in the study included (1) no active hepatitis C infection, (2) HIV co-infection, (3) hepatitis B virus co-infection, (4) active tuberculosis/on tuberculosis treatment, (5) eGFR <30 mL/min/1.73m^2^, (6) pregnancy, and (7) taking medications with serious drug-drug interactions with sofosbuvir/daclatasvir, which that participant is unable/unwilling to cease.

Participants with decompensated cirrhosis are eligible to access therapy through the study under the supervision of a consultant hepatologist if recommended after a consultant review. Consultant hepatologists are available on the weekends at the MLF clinic to see participants from the Burnet and MLF clinics; participants must attend the MLF clinic or Yangon Specialty Hospital to complete a specialist review. Advice from the consultant hepatologist is available via phone call and, if required, at other times.

### Study Assessments

**Figure 1:**
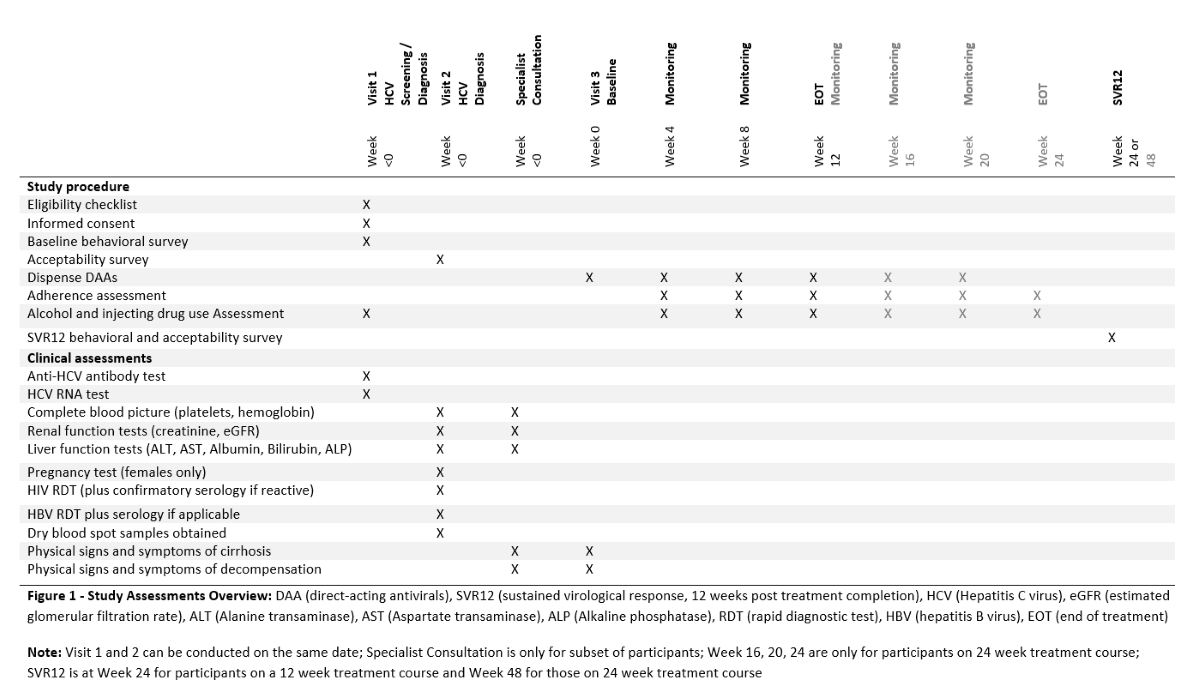
Study assessments overview.

**Figure 2:**
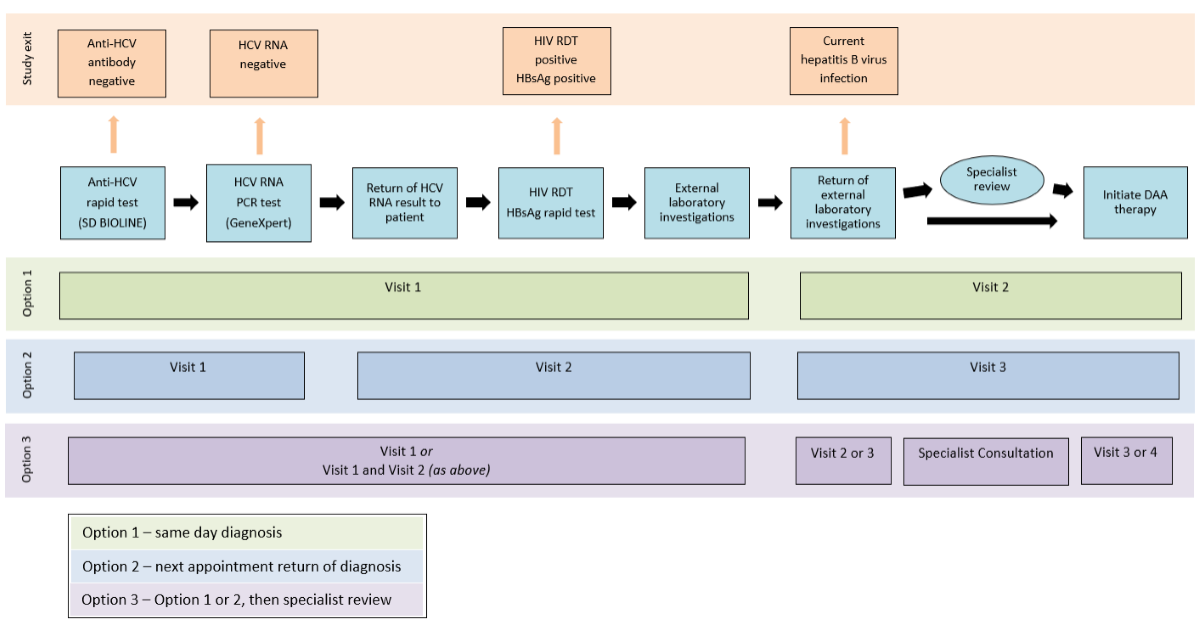
Study Visit Schedule.

Following consent and study enrollment, participants completed assessments, as outlined in the schedule of assessments (Figure 1). The visit schedule is described in Figure 2.

#### Visit 1: Screening and Diagnosis

This visit included a POC anti-hepatitis C Ab rapid test and POC hepatitis C RNA test, followed by an HIV Ab rapid test and a HBsAg rapid test. Blood samples were sent for pretreatment assessment investigations (further details given in *Pretreatment Assessment*). The hepatitis C RNA test result was returned to the participant during visit 1 or at the next appointment (visit 2).

#### Visit 2: Diagnosis (Return of Hepatitis C RNA Test Result)

If participants do not wait to receive the POC hepatitis C RNA test result during visit 1, they returned to the clinic to receive it at the next appointment.

Participants who tested hepatitis C RNA not detected were classified as having cleared their virus spontaneously (participants are not eligible if previously treated). They were informed of their results and do not require treatment.

#### Visit 2/3: Baseline (Pretreatment Assessment and Direct-Acting Antiviral Initiation)

##### Pretreatment Assessment

Pretreatment assessment included the following laboratory investigations:

- HIV Ab test (Alere Determine HIV-1/2 Ab rapid diagnostic test; performed at the study site)
- Hepatitis B surface antigen test (Alere Determine HBsAg rapid test performed at the study site)
- HBsAb test and HBcAb serology; hepatitis B DNA test performed if required
- Liver function tests: alanine aminotransferase (ALT), aspartate transaminase (AST), bilirubin, albumin, alkaline phosphatase
- Full blood examination: platelets and hemoglobin
- Renal tests: creatinine and eGFR.

These investigations were performed by a laboratory technician and at an external laboratory after RNA test results were available and returned to the participant at the next appointment (visit 2/3).

##### Cirrhosis/Decompensation Assessment

The APRI score was calculated to assess if liver cirrhosis was present. The medical officer calculated the APRI using the formula APRI=([AST/ULN]/ ([Platelet count × 10^9^/L])) × 100 [24], where ULN is the upper limit of normal. The AST ULN was set at 40 U/L for consistency across the study because laboratory reporting can vary.

The medical officer performed physical examinations for clinical signs of cirrhosis, including (1) liver enlargement with a firm liver edge, (2) spider nevi, (3) jaundice, (4) palmar erythema, (5) leukonychia, (6) gynecomastia, and (7) proximal muscle wasting/generalized sarcopenia. The medical officer also performed physical examinations and took a history of the clinical symptoms and signs of decompensation, including ascites, hepatic encephalopathy, and hematemesis and melena.

##### HIV and Hepatitis B Virus Co-Infection

Participants with HIV infection and chronic or acute hepatitis B infection were not eligible to commence treatment as part of the study. Participants with HIV or hepatitis B co-infection were referred to government-led QuickStart program hospital sites for further assessment of eligibility for this program or to the MLF clinic for other research studies or cost-sharing programs. Participants were referred to a local HIV treatment center if newly diagnosed.

##### Referral to a Hepatologist

Participants with (1) ALT or AST >200 U/L, (2) bilirubin above ULN (1.14 mg/dL), (3) albumin <35 g/L without other obvious cause, (4) jaundice, (5) ascites, (6) hepatic encephalopathy, or (7) hematemesis and melena were referred to a hepatologist for review.

Participants with ALT or AST levels between 100 and 200 U/L were advised to reduce alcohol intake, and the medical officer could consider locally available liver-supportive supplements as per the hepatologist’s advice. Liver function tests (LFTs) were retested after 2 weeks, with updated LFT results to inform if a referral to a hepatologist was required, and the updated APRI score was used to inform the treatment duration.

If the participants were identified with hepatic decompensation, they were referred to a specialist for care.

#### Direct-Acting Antiviral Therapy Initiation

DAAs were initiated by the study medical officer during visit 2/3 after reviewing and returning pretreatment assessment results to the participant. If the participant required a specialist review, DAAs were initiated (when appropriate) by the medical officer after the participant returned from the specialist review appointment.

#### On-Treatment Monitoring Visits

Participants commenced DAA therapy and received short on-treatment monitoring visits every 4 weeks (weeks 4, 8, and 12 for a 12-week course, plus weeks 16, 20, and 24 for a 24-week course). These visits include medication dispensing and questions about alcohol use, injecting drug use, medication adherence, and side effects. On-treatment visits are not required by the National Guidelines; they are included to collect study-related data.

#### Sustained Virologic Response 12 Assessment

After 12 weeks of treatment completion, the participants return to the clinic for the assessment of SVR12 using the Cepheid GeneXpert hepatitis C RNA test. If hepatitis C RNA is detected but <10 IU/mL, then the participant is retested in 12 weeks. If RNA is detected and within or above the quantitative range, the participant undergoes further testing using dried blood spot samples collected at screening and SVR12 to determine the next potential treatment course.

### Data Sources

#### Clinical Case Report Forms

Clinical data are collected in case report forms (CRFs) using the electronic database Open Medical Records System (OpenMRS); medical officers at the study site complete electronic CRFs (eCRFs) in OpenMRS first, but paper CRFs were used at the beginning of the study before eCRFs were finalized and are available if there are extended power outages or the database does not function properly. OpenMRS is an open-source medical records system; the database and the system are tailored to meet disease- and clinic-specific requirements. The OpenMRS database is run from a secure local server at each study site; backups of participant data are encrypted. Regular de-identified data exports are emailed to the data management team as a CSV (comma-separated values) file.

#### Visit 1/2 (Screening and Diagnosis): Case Report Forms 1 and 2

At visit 1/2 (Screening and Diagnosis), demographic data, drug use data, medical history, hepatitis C test results, and when participants received their diagnosis results were recorded.

Demographic data include age, sex, education level, employment status, income level, and residence locality. Drug use data include a history of injecting drug use, frequency of injecting, substances injected, receptive sharing of injecting equipment, history of and current methadone maintenance therapy, and alcohol consumption via the Alcohol Use Disorders Identification Test – Consumption (AUDIT-C) questionnaire.

#### Visit 2/3 (Baseline): Case Report Form 3 and Visit 3 /4 (Specialist Review) Case Report Form 7

At visit 2/3 (Baseline), pretreatment assessment laboratory results, clinical review, and treatment plan were recorded in eCRFs. A specialist review is captured in an eCRF by the medical officer when the participant returns to the study site, including laboratory investigation results, examination notes, and the recommended treatment plan.

#### On-Treatment Monitoring—Case Report Form 4

At monitoring visits (on treatment, end of treatment), the following data are collected: alcohol use in the past month using the adapted AUDIT-C questionnaire, injecting drug use in the past month (including frequency of injecting, substance/s most commonly injected), receptive sharing of injecting drug use equipment, current methadone maintenance therapy, side effects experienced, medication adherence (any missed doses, how many missed doses, and whether the doses were missed on consecutive days), and any new medications.

#### Sustained Virologic Response 12 Weeks Post-Treatment Completion Assessment: Case Report Form 5

At SVR12 visit (12 weeks after treatment completion), the following data are collected: alcohol use via the AUDIT-C questionnaire, injecting drug use in the past month (including frequency and substance), receptive sharing of injecting drug use equipment, current methadone maintenance therapy, hepatitis C RNA test result (SVR12 assessment), and details on when the participant received the result (on the same day or the next appointment).

#### Study Discontinuation: Case Report Form 6

When a participant exits the study, the date of exit and the reason for discontinuation (ineligible, loss to follow-up, participant decision, etc) are recorded. Participants are contacted at least three times over 4 weeks by phone and via their secondary contact at least once. A peer worker from the Burnet clinic also tries to contact the participant via outreach work. If there is no response or the participant is incarcerated, they are classified as lost to follow-up; if the participant has withdrawn from the study, this decision and the reason are recorded.

#### Participant-Completed Questionnaires

Participant-completed questionnaire data are collected in Research Electronic Data Capture (REDCap) [25]. REDCap is a web-based database system that hosts customized project-specific questionnaires. All participants completed a behavioral questionnaire at the first study visit (Screening visit). It collects data on demographics, lifetime health care utilization (locations and providers), alcohol use, injecting drug use, incarceration history, sexual behaviors, experiences of stigma/discrimination, and reason for seeking a hepatitis C test.

All participants also complete an acceptability survey at the Screening/Diagnosis visit, after receiving the results of anti-hepatitis C Ab and/or hepatitis C RNA tests. It collects information on referral to the clinic, how they found out about the clinic, how they got to the clinic (including transport mode, time spent, and cost), level of comfort in talking with a health provider about risk behaviors, confidence in the knowledge of hepatitis C, hepatitis C knowledge, acceptability of the Ab rapid diagnostic test (including the collection of blood samples, trust in test accuracy, and wait to receive results), and acceptability of POC GeneXpert hepatitis C RNA test (including the collection of blood samples, trust in test accuracy, and wait to receive results) using a 5-point Likert scale for acceptability and confidence, and testing preferences by asking which test option participants would choose.

Participants complete a combined behavioral and acceptability survey at the SVR12 visit. The survey collects data on the past months’ health care utilization, alcohol use (modified AUDIT-C questions for the past month), injecting drug use, acceptability of the testing and treatment process, details of how often participants attended the clinic, any costs to the participant, confidence in the knowledge of ongoing liver-related care required, and how to prevent reinfection.

#### Participant Interviews

Semistructured interviews will be conducted with 10 to 15 participants from each study site. The interview guide includes questions related to the experiences and views participants have toward services at the clinic, POC hepatitis C testing (both Ab and RNA testing), and treatment course. These data complement the acceptability survey data.

#### Provider Interviews, Surveys, and Clinical Workflow Observations

Semistructured interviews will be conducted with providers from each study site. Provider interviews will ask about the processes involved in conducting the tests, the processes involved in providing treatment, the referral process, and the processes for following up participants. These data will contribute to assessing the feasibility of the model of care from the provider perspective, contributing to domains of practicality and implementation. The provider survey will be completed by providers at each study site and will cover the acceptability and usability of Ab and RNA test devices and procedures. Clinical workflow observations recording workflow and time taken by staff members to complete specific aspects of workflow were completed midway through the study. These workflow observations will contribute to intervention complexity assessments.

#### Document Review

A document review of the GeneXpert device requirements, DAA requirements, training requirements, project meeting minutes, and monitoring visit reports will be used to inform intervention complexity assessments and practicality and implementation feasibility domains. GeneXpert device error reports and maintenance reports will also be included in the feasibility and intervention complexity assessment.

### Data Management

All quantitative CRF and survey data are managed in Stata SE 15 (Stata Statistical Software: Release 15, StataCorp LLC). OpenMRS and REDCap CSV file exports are imported into Stata and labeled and cleaned before conducting data quality checks and analysis. Regular data quality checks (every 2-4 weeks) and subsequent timely query resolution (<14 days) are performed to ensure quality assurance by the Burnet Institute project staff. The list of study IDs from the enrollment log is compared with OpenMRS and REDCap exports to ensure that no records are missing. Key data, including visit dates, test dates, test results, DAA therapy regimen and commencement date, and study discontinuation information, are checked for completion and logical responses.

Interviews will be audio recorded, transcribed, and translated into English. Transcript data will be managed using NVivo (QSR International).

### Statistical Analysis

#### Primary Outcomes

Outcomes 1-4 measuring the proportion of uptake across the cascade of care will be used to assess the feasibility of the model of care in terms of demand and practicality.

##### Outcome 1: The Proportion of Anti-Hepatitis C Antibody-Positive Participants who Received a GeneXpert Hepatitis C RNA Polymerase Chain Reaction Test

Descriptive statistics of the number and proportion of anti-hepatitis C antibody-positive participants who receive a GeneXpert hepatitis C RNA PCR test are provided. Any difference between sites will be determined using chi-square tests.

##### Outcome 2: The Proportion of Hepatitis C RNA Polymerase Chain Reaction-Positive Participants who Are Initiated on Direct-Acting Antiviral Therapy

Descriptive statistics of the number and proportion of hepatitis C RNA PCR-positive participants who are initiated on DAAs are provided. Any difference between sites will be determined using chi-square tests.

##### Outcome 3: The Proportion of Participants Initiated on Treatment who Complete Therapy

Descriptive statistics of the number and proportion of participants initiated on treatment who complete the therapy (defined as picking up the last 28-day bottle of medication and reporting ≤7 days missed doses) are provided. Any difference between sites will be determined using chi-square tests.

##### Outcome 4: The Proportion of Participants who Completed Treatment who Achieve Sustained Virologic Response 12

SVR12 is defined a priori as an undetectable hepatitis C RNA viral load using hepatitis C RNA PCR testing on the GeneXpert platform at least 12 weeks after therapy completion. Intention-to-treat SVR is defined by undetectable HCV RNA among all participants initiating treatment. Modified intention-to-treat SVR is calculated for participants who initiated treatment and have SVR data available (ie, the SVR test was performed, and the results are available).

Care cascade outcomes (primary outcomes 1-4) describe the proportion who have completed diagnosis, pretreatment assessments, and initiation of DAA therapy; completed DAA therapy; and achieved SVR12. These care cascade outcomes will be descriptively compared with historical outcomes to give context to these results in the study results paper.

#### Secondary Outcomes

##### Outcome 5: The Proportion of Participants Requiring Specialist Review

Descriptive statistics of the number and proportion of RNA-positive participants who require specialist review before commencing DAA therapy will be presented in aggregate format and by site. The reasons for specialist review and the outcome of the review will be presented.

##### Outcome 6: The Feasibility of the Testing and Treatment Pathway From the Provider Perspective

Interview data will be thematically and inductively analyzed using the intervention complexity framework to assess the feasibility and implementation requirements of the model of care from the provider perspective. This will be complemented by a document review of project documentation.

The framework includes (1) the characteristics of the interventions (device: GeneXpert device, cartridges; DAA prescription: DAA import, storage, and prescribing), (2) characteristics of delivery (facility and human resource requirements), (3) government capacity requirements (regulations and registrations), and (4) usage characteristics: ease of use, error rates and causes, and pre-existing demand.

##### Outcome 7: The Acceptability of the Testing and Treatment Pathways Among Participants

Descriptive statistics will be reported for measures of the acceptability of the testing and treatment pathways among participants and preferences for testing and treatment pathways. Categorical data will be reported using numbers and proportions in aggregate and by site. Questions using 5-point Likert scales will be collapsed into dichotomous variables, where appropriate for ease of reporting. Participant interview data will be analyzed using the theoretical framework for acceptability by Sekhon et al [26] and using inductive thematic analysis to identify any themes not captured by the framework analysis.

##### Outcome 8: Costing of the Point-of-Care Testing and Treatment Pathways at the Community Site

An ingredients-based costing method will be used to record the cost of consumables, staff time, and fractional costs of infrastructure and overheads for each person treated.

##### Outcome 9: Time From Hepatitis C RNA Test/Diagnosis to Treatment Initiation

The mean or median number of days and SD or IQR (depending on normality of results) of time from hepatitis C RNA test run date to treatment initiation will be presented in an aggregate format and by site.

### Trial Management

#### Reporting of Adverse Events

Adverse events are recorded in the participant adverse event report form, which collates adverse events per participant across the study. Serious adverse events are recorded in the serious adverse event report form and reported immediately to the study team, the study sponsor (FIND), the ethical review boards, and relevant local authorities/distributor companies if necessary.

#### Trial Monitoring Procedures

The Burnet Institute project coordinator makes monthly site monitoring visits and uses a site monitoring checklist covering completion of enrollment log, informed consent forms (correct version, signed and dated by participants and study staff), and observation of consent procedure (monthly only). The coordinator verifies that stock is stored correctly and sufficient stock is available to continue the study. Sponsor-led site monitoring visits are performed ad hoc by the FIND hepatitis C projects trial manager to verify informed consent forms and source data. The Myanmar Department of Medical Research Ethics Review Committee performs ad hoc monitoring visits, with two visits completed in the first 6 months. This study is not a safety/efficacy trial—it uses approved interventions, so it does not have a data safety monitoring board.

## Results

### Research Ethics Approval

Ethics approval was obtained from the Myanmar Department of Medical Research Ethics Review Committee (no. 2019-144) in December 2018 and the Alfred Hospital Human Research Ethics Committee (no. 244/17) in July 2017. All participants sign written informed consent forms.

### Project Status

As of September 30, 2019, 634 participants were enrolled in the study. All SVR12 data will be available by September 30, 2020, with results expected to be published by December 2020.

## Discussion

Globally, international and national guidelines support the implementation of decentralized community-based, GP-led models of care for hepatitis C [7,17]. However, few publications document such models of care in detail or their outcomes regarding the completion of the diagnostic pathway and treatment uptake and outcomes [12-14,19]. This study allows us to assess the implementation requirements of this clinical pathway, its feasibility, acceptability to both patients and providers, and its cost, along with the effectiveness of providing decentralized testing and treatment to patients in terms of the uptake of treatment and achievement of cure among those diagnosed. These results will inform the scale-up of services in Myanmar and other resource-constrained settings. In Myanmar, this will be particularly useful in informing the scale-up of services established to reach PWID who are not accessing the hospital-based program.

The study design has several limitations. It includes only two study sites in one large city, where amenities are more available than in more remote locations. Convenience recruitment, including a high proportion of study participants with known anti-hepatitis C Ab-positive test results, indicates that the study sample is not representative of case finding for hepatitis C infection. In addition, we were unable to treat people with HIV/hepatitis C co-infection; we aim to address this in further research studies in this setting.

Previous studies have demonstrated that treating people with hepatitis C infection with DAAs in community settings is feasible and that people achieve similar SVR12 rates to those treated in tertiary settings [12]; however, there is still little evidence for the feasibility and effectiveness of this model of care in low- and middle-income settings [13,14,19]. Decentralizing diagnostic testing and treatment initiation and offering these services at a one-stop shop has the potential to reduce time to DAA initiation, increase retention in care, and provide curative treatment to many people unable to access tertiary hospital services.

## Abbreviations

Ab: antibody
ALT: alanine aminotransferase
APRI: aspartate aminotransferase to platelet ratio index
AST: aspartate aminotransferase
AUDIT-C: Alcohol Use Disorders Identification Test – Consumption
CRF: case report form
CSV: comma separated value
CT2 Study: Hepatitis C: Community-based testing and treatment Study
DAAs: direct-acting antivirals
eCRF: electronic case report form
eGFR: estimated glomerular filtration rate
FIND: Foundation for Innovative New Diagnostics
GP: general practitioner
HBcAb: hepatitis B core antibody
HBsAb: hepatitis B surface antibody
HBsAg: hepatitis B surface antigen
HEAD-Start: Hepatitis C Elimination through Access to Diagnostics
MLF: Myanmar Liver Foundation
OpenMRS: Open Medical Records System
PCR: polymerase chain reaction
REDCap: Research Electronic Data Capture
SVR12: sustained virologic response 12 weeks after treatment completion
ULN: upper limit of normal
